# Fermented Soybean Meal (FSBM) in African Catfish (*Clarias gariepinus*) Diets: Effects on Growth Performance, Fish Gut Microbiota Analysis, Blood Haematology, and Liver Morphology

**DOI:** 10.3390/life12111851

**Published:** 2022-11-11

**Authors:** Muhammad Khairulanam Zakaria, Zulhisyam Abdul Kari, Hien Van Doan, Muhammad Anamul Kabir, Hasnita Che Harun, Suniza Anis Mohamad Sukri, Khang Wen Goh, Wendy Wee, Martina Irwan Khoo, Lee Seong Wei

**Affiliations:** 1 Department of Agricultural Sciences, Faculty of Agro-Based Industry, Universiti Malaysia Kelantan, Jeli Campus, Jeli 17600, Kelantan, Malaysia; 2 Advanced Livestock and Aquaculture Research Group, Faculty of Agro-Based Industry, Universiti Malaysia Kelantan, Jeli Campus, Jeli 17600, Kelantan, Malaysia; 3 Department of Animal and Aquatic Sciences, Faculty of Agriculture, Chiang Mai University, Chiang Mai 50200, Thailand; 4 Science and Technology Research Institute, Chiang Mai University, 239 Huay Keaw Rd., Suthep, Muang, Chiang Mai 50200, Thailand; 5 Department of Aquaculture, Sylhet Agricultural University, Sylhet 3100, Bangladesh; 6 Faculty of Data Science and Information Technology, INTI International University, Nilai 71800, Negeri Sembilan, Malaysia; 7 Center of Fundamental and Continuing Education, Universiti Malaysia Terengganu, Kuala Nerus 21030, Terengganu, Malaysia; 8 Department of Chemical Pathology, School of Medical Sciences, Universiti Sains Malaysia, Kubang Kerian, Kota Bharu 16150, Kelantan, Malaysia

**Keywords:** fish meal replacement, fermented soybean meal, African catfish, growth performance, protein replacement, sustainable aquaculture

## Abstract

The study revealed the potential of fermented soybean meal (FSBM) as a fish meal (FM) replacement in African catfish (*Clarias gariepinus*) feed formulation. Five isonitrogenous diets (32% crude protein) were prepared with five different levels of FSBM as FM replacement, namely 0% FSBM (T1), 40% FSBM (T2), 50% FSBM (T3), 60% FSBM (T4), and 70% (T5). The experimental fish was given the formulated diet for eight consecutive weeks. At the end of the feeding trial, the fish were subjected to growth performance, blood parameters, blood chemical, liver histology, and gut microbiota assessment. The study findings demonstrated that the experimental fish that received the T2 diet exhibited significantly higher (*p* < 0.05) growth performance. Experimental fish that received diet T2 had significantly higher (*p* < 0.05) white blood cell (WBC) and significantly lower (*p* < 0.05) in terms of cholesterol (CHOL), albumin (ALB), globulin (GLOB), and total protein (TP). The replacement of FSBM to FM significantly affected liver morphology on the sinusoid, vacuole, nucleus, and erythrocytes. Gut microbiota composition analysis showed a significantly high abundance (*p* < 0.05) of *Akkermansia muciniphila* in the experimental fish that received the T2 diet. The gut microbiota indicates that the experimental fish is in a healthy condition. In conclusion, replacing 40% FSBM with FM in aquafeed could enhance *C. gariepinus* growth performance and health conditions.

## 1. Introduction

Fish is an affordable protein source for approximately 1 billion people worldwide, regardless of their socioeconomic status [1]. Fish consumption was reported to be 9.0 kg annually in 1961 and doubled to 20.5 kg in 2018 [1]. The rapidly increasing human population is the major catalyst for the expansion of the aquaculture industry to fulfil the market demand. As a result, aquaculture has become one of the fastest-growing industries, with an average annual growth of 10%, contributing to approximately 90% of the global food demand in the last 20 years [1,2]. In 2020, the aquaculture industry recorded 178 million tonnes in production, generating a total revenue of USD 265 billion [3]. Nevertheless, high feeding costs and inconsistent supply remain major challenges in aquaculture [2,4].

Feed is the main cost in an aquaculture operation, making up 30–70% of the total production cost [5]. Fish meal (FM) is an important protein source in animal feed and is widely used for ruminants, pets, and aquafeed. Overreliance on the FM as the primary protein source in the aquafeed formulation has led to high demand and overpricing. Therefore, numerous studies have been conducted in seeking fish meal replacement [6,7,8,9,10,11,12,13,14]. Currently, soybean meal (SBM) is a plant-based protein identified as a potential FM replacement due to the consistent supply, nutritional profile, and reasonable price [15]. Thus, SBM is a more sustainable alternative as a feed ingredient than FM [16].

Despite being a promising ingredient for animal feed, plant-based proteins such as SBM possess anti-nutritional factors (ANFs) [17,18] consisting of phytic acids, trypsin inhibitors, and antigenic protein that can hinder protein absorption in animals [19] and interfere with their digestive enzymes activities [20]. These ANFs have significant negative impacts on fish growth performance [21,22,23]. At present, solid-state fermentation (SSF) has been introduced to minimise ANFs in SBM. The SSF was used in bioprocessing to detoxify agricultural wastes through fermentation [2,22,24]. Furthermore, SSF reportedly improved the nutritional profile of SBM, and thus, was beneficial for animals [15] and aquaculture [25,26,27]. Previous studies indicated that fermented soybean meal (FSBM) was a better alternative ingredient that has the ability to improve growth and health performance than SBM in fish feed [28,29].

Generally, lactic acid bacteria (LAB), such as *Bacillus* spp. and yeast, are utilised in FSBM production [30]. In addition, the Staphylococci bacteria are regularly used in food fermentation. Staphylococci can be divided into coagulase-positive staphylococci (CPS) and coagulase-negative staphylococci (CNS). The CPS are pathogenic and responsible for food poisoning [31]. On the other hand, CNS is a common starter in cheese production [32], seafood [33], sausage [34], and soybean-based product processing [35,36]. For example, *Staphylococcus succinus* is a commercially used CNS in soybean fermentation [35]. Nonetheless, reports on *S. succinus* for FSBM production as animal feed remain lacking. In the current study, FSBM was designated as a model replacement material of FM. *S. succinus* was employed as a model bacterium to demonstrate this research into African catfish. To obtain optimum growth and health performance, the FSBM inclusion level should also be at optimum. Therefore, the study was carried out to determine the impact of FSBM replacement at different percentages (0%, 40%, 50%, 60%, and 70%) on the growth performance, blood haematology, liver morphology, and gut microbiota regulation in African catfish, *Clarias gariepinus*.

## 2. Materials and Methods

### 2.1. Research Ethic Approval

The present study was approved by the Faculty of Agro-based Industry Animal Ethics Committee, Universiti Malaysia Kelantan (File No: UMK/FIAT/ACUE/UG/14/2021) and complied with the National Institute of Health of Malaysia guidelines.

### 2.2. Experimental Fish Preparation

The experimental fish used in this study was prepared as described in a previous study [37]. A total of 500 African catfish fingerlings, approximately 10 cm in length and 7 g in weight, were purchased from a commercial farm located at Tanah Merah, Kelantan. The fingerlings were acclimatised for 2 weeks and fed a commercial pellet (Star mill, Malaysia) with a crude protein content of 30%. After the acclimatisation period, 300 healthy fish were selected for the feeding experiment. Each experimental tank (30 L) was stocked with 20 experimental fish that were provided with the formulated feed at a feeding rate of 5% of body weight daily. The fingerlings were fed twice daily (8 am and 3 pm), while 70% water changes were performed daily at 6 pm. The experiment was carried out for eight weeks, and the total length and weight of the experimental fish were measured weekly. Meanwhile, the water quality in the experimental tanks was measured and recorded once a week. The water parameters were as follows: pH = 7–7.6, dissolved oxygen = 5.6 to 6.2 mg/L, temperature = 26.5–28.8 °C, and ammonia ≤ 1 mg/L.

### 2.3. Preparation of Defatted SBM Protein Mixture

Live bacteria of *Staphylococcus succinus* were cultured in Tryptic Soy Broth (TSB) (Merck, Darmstadt, Germany) for 24 h at room temperature. The bacterial cells were harvested by centrifugation using a minispin (Eppendorf, Hamburg, Germany) and the concentration of bacterial cells was adjusted into 1 × 10^6^ CFU/mL using physiological saline. The defatted SBM protein mixture was prepared by adding 1 g of defatted SBM in 8 mL of distilled water. Subsequently, 1 mL of *Staphylococcus succinus* suspension (Source: FSBM; Isolation and identification as described by Thomson et al. [38]) was added to the mixture, followed by homogenisation and incubation at room temperature for one week. The mixture was homogenised and incubated at room temperature together with a mixture without bacterial inoculation as a control. All the experiments were carried out in triplicate. The prepared feed samples were subjected to soluble protein measurement (1 mL) followed the method by Shen et al. [39] at an hourly interval for 8 h to monitor the total soluble protein content.

### 2.4. Fish Feed Preparation

The experimental fish feed was prepared as described by Abdul Kari et al. [22]. Five types of fish feeds were prepared, namely T1, T2, T3, T4, and T5 as shown in Table 1. Fish meal and SBM acted as protein sources in the control group (T1), whereas fermented SBM (FSBM) was used as fish meal and SBM replacement for the other treatment groups. The total protein content in the fish feed of all treatment groups ranged from 31.34 to 33.44%. All raw materials were mixed homogenously and produced using a pellet extruder (diameter: 2–3 mm). The formulated feed was oven-dried at 40 °C for 24 h and refrigerated at 4 °C. In addition, all formulated diets were subjected to proximate analysis following the AOAC protocol described by Thiex et al. [40]. Meanwhile, the total amino acid of each formulated feed was performed as described by Kader et al. [41] as shown in Table 2. All the feed analyses in this study were performed in triplicate.

### 2.5. Growth Performance of Experimental Fish

The experimental fish was fasted for 24 h before euthanising them using clove oil at the end of the experiment. The samples were collected for growth performance analysis using the formulas recommended by Kari et al. [2]:

- Survival rate (%) = (Total number of survived fish/Total number of experimental fish at the beginning of the experiment) × 100%
- Weight gain (%) = (Final weight − initial weight) × 100%/initial weight
- Specific growth rate (%) = (log Final weight − log initial weight) × 100%/Experiment duration
- Hepatosomatic (%) = (Weight of liver/body weight) × 100%
- Visceral somatic (%) = (Visceral weight/body weight) × 100%
- Feed conversion rate (FCR) = Total feed consumption/Fish weight gain

### 2.6. Fish Gut Microbiology Analysis

The total bacteria and *Staphylococcus succinus* colonies in the fish samples were determined via the 10-fold dilution method as described by Seong Wei et al. [43], using Tryptic Soy Agar (TSA) (Merck, Germany) and mannitol salt agar (Himedia, Mumbai, India), respectively. First, the inoculated medium was incubated for 24 h at room temperature. Subsequently, the total bacterial colonies were recorded as colony-forming units (CFU)/g of the fish gut. The experiment was conducted in triplicate.

### 2.7. Fish Blood Haematology and Biochemical Analysis

Blood haematology and biochemical assay for the fish samples were according to He et al. [44]. First, three fish were randomly sampled in each treatment group and anesthetised using clove oil. The insulin syringe was then used to draw the fish’s blood and store it in heparinised tubes. In the blood haematology analysis, 150 µL of fish blood from each group was subjected to a blood count test using the automatic hematology analyser (Mythic 18 Vet, Cormay, Fort Lauderdale, FL, USA). Meanwhile, another 150 µL of fish blood samples characterised their blood biochemical properties. Finally, the blood sample was dropped on cassettes (Idexx, Westbrook, ME, USA), and the results were visualised by the VetTest analyser (Idexx, USA).

### 2.8. Histological Analysis

The histological analysis was conducted as described by Lee et al. [45] and Abdul Kari et al. [22]. At the end of the feeding trial, the fish liver tissue from each treatment group was sampled and examined under the light microscope (Leica, Wetzlar, Germany) at 40× magnification. Abnormalities found in the samples were visualised using imaging software (Cellsens software, The Hague, The Netherlands) to evaluate the presence of sinusoids, vacuole, nucleus, and erythrocytes.

### 2.9. Next Generation Sequence (NGS) Metagenomics Data Analysis

#### 2.9.1. Fish DNA Preparation

The DNA extraction from the fish gut samples was performed following Miler et al. [46] and Inglis et al. [47]. First, the samples were centrifuged (10,000× *g*) to obtain the pellet, followed by washing with sorbitol buffer. The samples were resuspended using the homogenisation buffer, placed in silica beads (0.1 mm) microtubes (1.5 mL), and vortexed at 4000 rpm for 30 min. Subsequently, the suspension was subjected to protein precipitation for 5 min at 0 °C before centrifugation (10,000× *g*) for 10 min. Isopropanol was added to the supernatant (1:1) and centrifuged (10,000× *g*) for 10 min to precipitate the DNA pellet. Finally, 75% ethanol was used to wash the DNA pellet twice prior to resuspension in 0.1 × TE buffer. The DNA sample was stored at −20 °C until use.

#### 2.9.2. Library Data Preparation

The amplification of bacterial 16s rRNA V3 hypervariable region from provided gDNA was performed using the following primers: 341F: CCTACGGGNGGCWGCAG and 518R: ATTACCGCGGCTGCTGG following the methods of Klindworth et al. [48] and Garcia-Lopez et al. [49]. Five additional bases were included at the 5′ end of the primers for inline barcoding [50]. Subsequently, the polymerase chain reaction (PCR) amplification protocol was executed as follows: 3 min of DNA denaturation at 95 °C, 30 s of DNA denaturation at 95 °C for 28 cycles, 20 s primer annealing at 55 °C and 10 s strand extension at 72 °C. Finally, the gel electrophoresis was performed to visualise the barcoded PCR products, followed by normalisation and pooling based on the intensity and purified with 1.5× vol of SPRI bead.

#### 2.9.3. DNA Sequencing Assay

The purified pooled amplicons were processed to incorporate the Illumina adapter and dual index barcodes using the NEB Ultra II Library preparation kit. The constructed library was quantified with Denovix high-sensitivity assay and sequenced on an iSeq100 (Illumina, San Diego, CA, USA) for 2 × 150 paired-end sequencing.

#### 2.9.4. Data DNA Sequencing Analysis

Fastp v0.21 was used to overlap paired-end reads, and Cutadapt v1.18 was applied to remove the merged reads, as per Chen et al. [51]. Reads that have been demultiplexed and trimmed were then imported into QIIME2 v.2021.4 and denoised using dada2 as described by Bolyen et al. [52] and Callahan et al. [53]. The Q2-feature-classifier was used to taxonomically assign and train the ASV by feeding the updated GTDB release r202 16s rRNA data, consisting of 254,090 bacterial and 5316 archaeal genomes [54,55]. The ASVs were then subjected to further analysis. The ASV and taxonomic classification tables were exported into tab-separated values using QIIME 2 tools to produce Microbiome Analyst—compatible input described by Chong et al. [56]. The input was utilised in establishing the SparCC co-occurrence network, as described by Friedman and Alm [57]. Meanwhile, the linear discriminant analysis (LDA) effect size (LEfSe) data was statistically analysed [58]. Furthermore, the QIIME2 plugins were used to determine the Alpha- and beta-diversity. The filtered relative abundance table was also used as the input to generate Krona plots for the intuitive exploration of relative abundances within the hierarchies of taxonomic classifications [59]. In addition, only clusters with an accumulated relative abundance of more than 1% and sample prevalence of more than 20% were utilised for heatmap generation.

### 2.10. Statistical Analysis

All the collected data were tested for normality before further analysis. First, Levene’s test examined the variance homogeneity of data to confirm the normality and homogeneity of data. The statistical analysis of this study was performed using the Statistical Package for the Social Sciences (SPSS) version 26.0. Specifically, the fish growth performances were evaluated using a one-way analysis of variance (ANOVA) followed by the Tukey post hoc test. The results were presented as mean ± SD at a significant level of *p* < 0.05.

## 3. Results

### 3.1. Fish Growth Performance

Table 3 presents the fish growth performance after the feeding trial. There were significant differences (*p* < 0.05) for all growth performance parameters. The fish in the T2 diet group demonstrated the best final weight (245.5 ± 8.35%), weight gain (2283.8 ± 104.21%), and SGR (2.46 ± 0.034%). In addition, the T2 group recorded the lowest visceral somatic index (2.85 ± 0.278), hepatosomatic index (1.15 ± 0.047), and FCR (1.06 ± 0.038%). Conversely, the fish in the T5 diet group exhibited the highest (*p* < 0.05) visceral somatic Index (4.13 ± 0.147%), hepatosomatic index (1.47 ± 0.084%), and FCR (1.27 ± 0.033). Notably, no mortality was recorded for all treatments throughout the study.

### 3.2. Fish Gut Microbiology Analysis

Total bacteria and *S. succinus* in the fish gut were recorded in Table 4. The highest total bacteria was found in the fish gut of the T5 group (3.67 ± 0.110 × 10^9^ CFU/g). Meanwhile, the highest total *S. succinus* was recorded in the gut of experimental fish that received a T2 diet (4.03 ± 0.164 × 10^6^ CFU/g).

### 3.3. Blood Parameters Analysis

Table 5 exhibits the blood parameters of fish fed with different diets. The group that was provided with the T2 diet demonstrated significant (*p* < 0.05) and highest white blood cell (WBC) count, followed by the control and T3 diet group. Conversely, the fish provided with T5 diet demonstrated significant (*p* < 0.05) and the lowest WBC count. Meanwhile, the T2 group recorded significant (*p* < 0.05) and the highest lymphocytosis. In contrast, all groups observed no significant difference in monocyte (MON) count. Furthermore, the lowest blood granulocytosis (GRA) (*p* < 0.05) was observed in the T2 diet fish samples. On the other hand, the control group (T1) exhibited the highest GRA, followed by T5, T4, and T3 groups. In addition, a significant variation was observed in the red blood cell (RBC) count, where the significant and highest (*p* < 0.05) RBC was found in the T2 group. Moreover, the haemoglobin (HGB) level, mean corpuscular haemoglobin concentration (MCHC), red cell distribution width (RDW), platelet (PLT), mean platelet volume (MPV), and platelet distribution width (PDW) were significantly different between treatments, without any specific trend. On the contrary, no significant differences were observed in the haematocrit (HCT), mean corpuscular volume (MCT), and procalcitonin (PCT) among the treatment groups.

### 3.4. Blood Biochemical Parameters

Table 6 presents the blood chemical profiles of the different experimental groups. The groups supplemented with FSBM recorded significantly lower (*p* < 0.05) albumin (ALB), globulin (GLOB), total protein (TL), cholesterol (CHOL), and glucose (GLU). Meanwhile, the urea (BUN), creatinine (Crea), alkaline phosphatase (ALKP), gamma glutamyltransferase (GGT), and total bilirubin (TBIL) of all groups were relatively similar. Furthermore, the alanine aminotransferase (ALT) and aspartate aminotransferase (AST) were significantly lower in the control (T1) and T2 groups compared to T3, T4, and T5.

### 3.5. Experimental Fish Liver Histological Analysis

**Figure 1:**
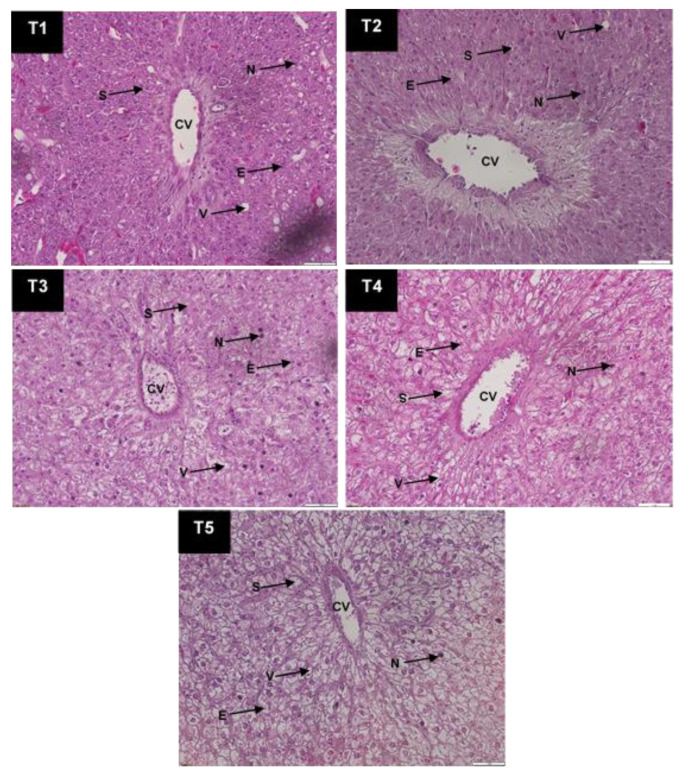
Histological morphology of the African catfish liver fed with FSBM at different inclusion levels as seen under the light microscope at 40× magnification (Olympus BX43). Changes were evident in the sinusoid (S), vacuole (V), nucleus (N), erythrocytes (E), and central vein (CV); Scale bar: 200 μm.

The liver histomorphology of the experimental fish is presented in Figure 1. There were changes in liver cells at varying levels, involving the sinusoid, vacuole, nucleus, and erythrocytes with different FSBM diets. For instance, the liver cells of fish that were provided with a 40% FSBM diet exhibited better nuclei and cytoplasm in terms of arrangement and structure than other treatment groups. Nevertheless, the nuclei and cytoplasm were atrophied, while the hepatic cell cords were disorganized in groups T4 and T5. In addition, the vacuolar cytoplasm increased with FSBM inclusion level; but this was not the case for the T1 and T2 groups.

### 3.6. Experimental Fish Gut Microbiota Analysis

**Figure 2:**
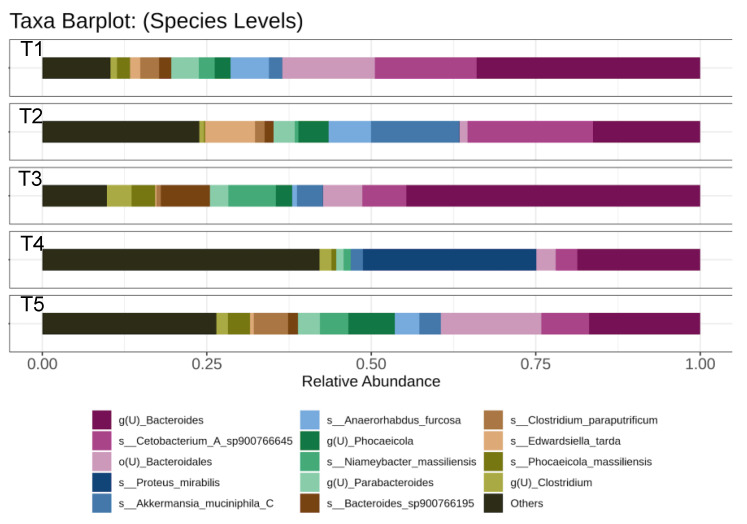
The abundance of gut microbiota in experimental fish.

Figure 2 illustrates the abundance of gut microbiota in all experimental groups. Overall, 15 groups of gut microbiota were identified in this study. *Bacteroides* were significantly present, and the highest in fish fed with the T3 diet, followed by control (T1). Meanwhile, the presence of *Bacteroides* was comparable in T2, T4, and T5 groups. Furthermore, *Proteus mirabilis* was significantly detected and the highest in the gut of T4 fish, whereas none or small amounts were observed in other treatment groups. *Akkermansia muciniphila* was significant, and the highest in the fish fed with the T2 diet, while the other treatment groups exhibited a similar abundance of this microorganism. *Anaerorhabdus furcosa* was significantly higher in T1 and T2 groups compared to T3 and T5. However, this gut microbiota was not found in T4 fish. Meanwhile, *Phocaeicola massiliensis* was significant and the highest in the T3 group, followed by T5 and T3. None or a small amount of gut microbiota was detected in other treatment groups. Finally, the highest abundance of *Edwardisiella tarda* was recorded in the T2 treatment group.

## 4. Discussion

The current study evaluated the effects of FSBM at different inclusion levels on *C. gariepinus* growth performance and health. Several analyses were performed in this study, including fish gut microbiology analysis, blood biochemistry, next-generation sequencing (NGS), metagenomics data analysis, and liver histology. African catfish, *C. gariepinus,* was selected for this study because it is an important aquaculture species and economically important worldwide, adaptable to various conditions, can be farmed in high-density facilities, has rapid growth, and has good quality meat for human consumption. It was reported that biotechnological processes, such as FSBM as a protein supplement, can improve SBM nutritional quality for carrying probiotics and other plant feedstuffs [2,60,61]. In this study, FSBM can be included up to 40% in the African catfish diet without adverse effects on growth performance (245.5 ± 8.35 g) compared to other treatments (0, 50, 60 and 70% FSBM) after eight weeks. Several studies reported that FSBM is a promising FM replacement and improved the growth performances of various species, namely orange-spotted grouper, *Epinephelus coioides* [27], rainbow trout, *Oncorhynchus mykiss* [25], yellowtail, *Seriola quinqueradiata* [62], black sea bream, *Acanthopagrus schlegelii* [63], pacific white shrimp, *Litopenaeus vannamei* [64], and piglets [65]. Furthermore, Novriadi [66] concluded that more than 40% FSBM inclusion level could decrease the growth performances of farmed animals, consistent with the current study findings. The bioactive soy product in the fish diet act as a protein supplement and growth promoter for African catfish by enhancing the protein metabolic utilisation and delivery to the gut [2,18,22]. The benefits of FSBM as a growth promoter were demonstrated by the improvements in morphology body indices, biochemical, haematology, blood biochemical, and liver morphology variables in fish. In addition, the quality of plant ingredients used in aquafeeds is indicated by the growth performance, biochemical composition, immunological factors, and histopathology [67,68].

Earlier studies have reported that lactic acid fermentation can enhance the nutritional profile of soybean by-products by partially removing anti-nutritional factors (ANFs) and feed allergens, thus, improving the growth performance, digestibility, and physiology (bile status, intestinal microbiota) in the aquaculture species [2,22,69,70,71,72]. Furthermore, the highest weight gain (2283.8 ± 104.21g) and SGR (2.46 ± 0.034%) were obtained when the fish was fed with the 40% FSBM diet. Furthermore, the application of FSBM as an aquafeed has been widely reported. For instance, Li et al. [73] revealed that *Enterococcus faecium* FSBM improved the growth performance and immunity of turbot (*Scophthalmus maximus* L.). Likewise, Silva-Carrillo et al. [74] claimed that FSBM has enhanced the growth performance of juvenile spotted rose snapper *Lutjanus guttatus* (Steindachner, 1869). Additionally, FSBM is a potential FM replacement in the diet of largemouth bass, *Micropterus salmoides* [75], largemouth bass (*Micropterus salmoides*) [27], white shrimp (*Litopenaeus vannamei*) [76], and African catfish (*C. gariepinus*) [2,22].

The Hepatosomatic Index (HSI) is essential to measure an animal’s energy reserves, particularly fish. The present study demonstrated that increased FSBM inclusion in the fish diet corresponded with improved growth performance, contrary to the fish hepatosomatic index (HSI), which is directly correlated with the deposition of glycogen and lipids within the liver [77,78]. Similarly, the gilthead sea fed with fish meal-based diets supplemented with soybean by-products exhibited the same response [79]. In the current study, FSBM supplementation significantly impacted the HSI values, indicating that the energy reserves of fish varied between the experimental groups throughout the eight-week feeding trial. The lowest HIS value was recorded by the 40% FSBM group (1.15 ± 0.047%), consistent with Kari et al. [2], where fermented soy pulp was utilised as an FM replacement.

The fundamental biological ratio, such as the visceral somatic index (VSI), was evaluated in this study to assess the feed value [80]. The increase in FSBM percentage resulted in the rise of the VSI; the lowest VSI was obtained in fish fed with 40% FSBM feed (2.85 ± 0.278%), suggesting the high muscle composition of the fish and a desirable research outcome. Previously, it was reported that VSI increases with carbohydrate content [81], which contradicted the current study findings. The experimental feed was prepared as four isonitrogenous diets, with a carbohydrate composition ranging from 40% to 45%. In addition, FCR is crucial in estimating the feed required for the growing cycle of an aquaculture species, besides helping farmers evaluate the profit of their business. The FCR in the present study illustrated a decreasing trend for the experimental diet groups, except for T3, where the FCR significantly increased throughout the study period. Meanwhile, 40% FSBM feed fish recorded the lowest FCR (1.06 ± 0.038). All the FSBM groups contained approximately 32% crude protein and 4–5% lipid, resulting in FCR values of <2. This finding is supported by an earlier study that revealed the bioactive role of dietary probiotics in reducing the FCR [82]. Consequently, FSBM is a sustainable and cost-effective aquafeed ingredient, which can reduce the overall feeding cost that makes up 30–70% of the total production cost of an aquaculture farm [83].

Shiu et al. [84] reported that the histological status of the liver is a good indicator of the actual nutritional status of the fish. In the present study, FSBM supplementation contributed to histological, morphological, and functional changes in *C. gariepinus* liver tissues after eight weeks. Previously, studies have revealed the adverse effects of using SBM as a protein source in the aquafeed formulation. For instance, Heikkinen et al. [85] reported that SBM could lead to abnormal histological changes in the liver of juvenile rainbow trout liver, Atlantic salmon [86], *Oncorhynchus mykiss* [25], and *Psetta maxima* L. [87]. Nevertheless, histological alterations vary depending on fish species, feed formulation, and experimental conditions. Furthermore, ANFs such as b- conglycinin and glycinin in SBM could compromise digestion and nutrient absorption in animals [88]. Therefore, the ANFs in SBM were reduced via fermentation involving various microbial species that produced different by-products. For example, lactic acid bacteria (LAB) FSBM generates lactic acid and antimicrobial peptides [89], *Bacillus* FSBM yields antibiotic substances [90], and yeast FSBM produces β-glucan, vitamins, and organic acids [91]. Nonetheless, excessive FSBM in feed formulation may be hazardous as FSBM by-products such as acetic acid and biogenic amines (e.g., histamine) are harmful to animals [92]. Therefore, FSBM was added in aqua feed at low percentages (±5%) in China commercial fish feed. Furthermore, FSBM should not be exposed to high temperatures, which may inactivate the microbes and their metabolites [93].

In the present study, experimental fish in the T2 diet group exhibited significantly high WBC (132.9 ± 2.21) and RBC (2.78 ± 0.23 10^3^/µL). According to Taufek et al. [94] and Abdul Kari et al. [22], the high WBC is due to the increasing amount of antigen in the circulation system. High WBC and RBC in the circulatory system can increase oxygen transportation capacity and boost animal immune systems [22]. Moreover, the immunostimulatory effects of FSBM are evident due to the increased WBC in the T2 diet group, consistent with their growth parameters. Furthermore, the experimental diets did not affect the haematological values, which remained within the normal range for a healthy African catfish [16]. Thus, it can be concluded that FSBM supplementation boosted the haematological parameters in African catfish, leading to enhanced growth performance and health status. Similarly, Svobodová et al. [95] evaluated the nutritional status and feed composition of fish relative to the environmental conditions by referring to the haematological parameters. Additionally, WBC, RBC, and HCT are useful indicators of feed anti-nutritional toxicity and fish health [96]. Generally, the blood parameter values often vary depending on several factors, including rearing conditions and species [94,97,98]. Meanwhile, mean corpuscular haemoglobin concentration (MCHC) is the haemoglobin level in each RBC and can be utilised in assessing animal anaemia [22]. The MCHC value of <28 g/dL often indicates anaemia. The MCHC values in this study ranged between 28.7 to 33.2 g/dL for all groups, suggesting that all the experimental fish were not anaemic. Furthermore, albumin and globulin were significantly higher in the fish fed with the FSBM-supplemented diet, indicating the good health status of the experimental fish [99]. The blood parameters are also used to monitor liver and kidney diseases, whereas alanine aminotransferase (ALT) and aspartate aminotransferase (AST) are key indicators of stress and tissue impairment in animals [100]. The experimental fish supplemented with a high dose of FSBM recorded elevated ALT and AST levels, possibly compromising the health status of African catfish.

Numerous studies have recently performed high-end sequencing to discover the effects of FSBM on the fish gut microbiota. For example, Li et al. [73] revealed the effect of *Enterococcus faecium* FSBM on turbot, *Scophthalmus maximus* L. gut microbiota and Yang et al. [101] reported that yeast, *Saccharomyces cerevisiae,* and *Lactobacillus casei* FSBM modulated and enhanced the gut microbiota in juvenile barramundi, *Lates calcarifer*. In the present study, *S. succinus* MF 116251 FBSM at 40% inclusion improved the gut microbiota of African catfish, which was validated by the presence of *Akkermansia muciniphila* in abundance. *A. muciniphila* is a healthy biomarker bacterium present in the intestines of human and animals [102,103,104] and one of the next generation probiotics that indicates the good health status of an organism. Furthermore, *A. muciniphila* is essential for host immunity, gut health maintenance, and metabolic modulation [105,106]. The absence of this bacteria is often associated with diseases [107,108,109]. Therefore, *A. muciniphila* presence in the gut of the experimental fish that consumed 25% FBSM diet indicated their good health status. However, future studies should investigate other parameters, namely gut morphology, digestive enzyme, and the gene expression of transforming growth factor-beta 1 (TGF-β1), nuclear factor kappa-B (NF-kβ), heat shock protein (hsp90a), and lysozyme (lyzg), which are crucial in evaluating new feed efficiency. Moreover, future studies should assess the potential of *S. succinus* MF 116251 FSBM as a protein source for other aquatic animals.

## 5. Conclusions

In summary, FSBM is a promising FM alternative that could improve the growth performance, health, and liver morphology of African catfish. At 40% inclusion in the fish diet, FSBM enhanced the fish growth performance and health status. Furthermore, FSBM inclusion promoted the fish gut microbiota and improved several blood parameters in *C. gariepinus*. The study findings also revealed a decline in growth performance when the FSBM level exceeded 40%. In addition, this study will provide the baseline information for researchers to discover the full potential of *S. succinus* MF 116251 FSBM in the aqua-feed industry.

## Figures and Tables

**Table 1:** Composition and proximate analysis (g/100 g dry weight) of the experimental diets for *C. gariepinus*.

Ingredients (g/kg)	Diets (%)				
	T1	T2	T3	T4	T5
Fish meal ^1^	40	30	20	10	0
SBM	30	0	0	0	0
FSBM ^2^	0	40	50	60	70
Wheat	20	20	20	20	20
Fish oil	6	6	6	6	6
CMC ^3^	2	2	2	2	2
Vitamin premix ^4^	1	1	1	1	1
Mineral premix ^5^	1	1	1	1	1
Total	100	100	100	100	100
Proximate analysis (%)					
Protein	31.34	32.32	32.98	32.92	33.44
Carbohydrate	47.70	46.67	46.32	46.63	46.98
Lipid	5.50	5.21	5.01	4.78	4.63
Fibre	4.50	4.45	4.34	4.21	4.09
Ash	5.66	5.99	5.86	5.92	5.38
Moisture	5.30	5.36	5.49	5.54	5.48

^1^ Danish fishmeal; ^2^ Fermented soybean meal; ^3^ Carboxymethyl cellulose as a binder; ^4^ Vitamin premix (mg or IU/kg diet): vitamin A, 6000 IU; vitamin D3, 2000 IU; ascorbic acid, 200 mg; vitamin E, 50 mg; menadione, 5 mg; thiamine,15 mg; riboflavin, 15 mg; nicotinic acid, 30 mg; pantothenic acid, 35 mg; pyridoxine HCl, 6 mg; cyanocobalamin, 0.03 mg; biotin, 0.2 mg; inositol, 200 mg; folic acid, 3 mg; ^5^ Mineral premix (mg/kg diet): iodine, 0.4 mg; cobalt, 0.1 mg; copper, 4 mg; iron, 150 mg; zinc, 80 mg; manganese, 20 mg; selenium, 0.1 mg; magnesium, 100 mg.

**Table 2:** Total amino acid profile of formulated fish feed vs. African catfish requirement. Data expressed as mean ± standard deviation.

Amino Acid (%)	T1	T2	T3	T4	T5	(%)
Lysine	1.53 ± 0.04	1.51 ± 0.32	1.49 ± 0.04	1.46 ± 0.23	1.45 ± 0.28	1.43 *
Methionine	0.98 ± 0.03	0.82 ± 0.03	0.76 ± 0.04	0.73 ± 0.06	0.68 ± 0.04	0.64 *
Arginine	6.64 ± 0.32	6.31 ± 0.22	5.81 ± 0.54	5.51 ± 0.11	4.98 ± 0.32	1.20 *
Phenylalanine	1.23 ± 0.21	2.44 ± 0.32	2.56 ± 0.12	2.78 ± 0.45	3.68 ± 0.32	1.40 *

* African catfish nutritional requirement by National Research Council [42].

**Table 3:** Growth performance of African catfish fed with different FSBM percentage for eight weeks (*n* = 3). Data expressed as mean ± standard deviation.

Parameters	T1	T2	T3	T4	T5
IW (g)	10.3 ± 0.06	10.3 ± 0.10	10.2 ± 0.12	10.3 ± 0.06	10.2 ± 0.10
FW (g)	195.4 ± 14.47 ^d^	245.5 ± 8.35 ^a^	227.0 ± 4.80 ^b^	214.7 ± 3.88 ^b^	206.9 ± 5.03 ^c^
WG (%)	1791.1 ± 148.79 ^d^	2283.8 ± 104.21 ^a^	2118.4 ± 67.93 ^b^	1991.4 ± 49.50 ^c^	1928.3 ± 58.60 ^c^
SGR (%)	2.28 ± 0.061 ^d^	2.46 ± 0.034 ^a^	2.40 ± 0.024 ^b^	2.36 ± 0.018 ^c^	2.33 ± 0.022 ^c^
VSI (%)	3.59 ± 0.48 ^b^	2.85 ± 0.278 ^a^	3.53 ± 0.131 ^b^	3.69 ± 0.218 ^c^	4.13 ± 0.147 ^d^
HIS (%)	1.34 ± 0.183 ^b^	1.15 ± 0.047 ^a^	1.28 ± 0.071 ^c^	1.33 ± 0.118 ^b^	1.47 ± 0.084 ^d^
FCR	1.36 ± 0.106 ^d^	1.06 ± 0.038 ^a^	1.15 ± 0.026 ^b^	1.22 ± 0.023 ^c^	1.27 ± 0.033 ^c^

Note: Different superscripts showed significant differences at *p* < 0.05. Abbreviation: IW, Initial weight; FW, Final weight; WG, Weight gain; SGR, Specific growth rate; VSI, Visceral somatic Index; HIS, Hepatosomatic index; FCR, Feed conversion ratio.

**Table 4:** Total bacteria in fish gut. Data expressed as mean ± standard deviation.

Parameters	T1	T2	T3	T4	T5
Total bacteria CFU/g intestine × 10^9^	2.78 ± 0.454 ^a^	3.00 ± 0.498 ^b^	3.20 ± 0.225 ^b^	2.80 ± 0.427 ^a^	3.67 ± 0.110 ^c^
*Staphylococcus succinus* CFU/g intestine × 10^6^	1.58 ± 0.332 ^a^	4.03 ± 0.164 ^b^	2.98 ± 0.023 ^c^	1.70 ± 0.203 ^a^	2.77 ± 0.210 ^c^

Note: Different superscripts showed significant differences at *p* < 0.05.

**Table 5:** Fish blood parameters fed with different dietary FSBM protein supplement level (*n* = 3). Data expressed as mean ± standard deviation.

Blood Parameters	T1	T2	T3	T4	T5
WBC (10^3^/µL)	121.2 ± 3.23 ^a^	132.9 ± 2.21 ^b^	121.3 ± 2.31 ^a^	116.7 ± 1.42 ^c^	113.9 ± 2.31 ^d^
LYM (%)	89.4 ± 4.51 ^a^	109.8 ± 3.53 ^b^	98.5 ± 2.67 ^c^	96.7 ± 3.41 ^c^	97.8 ± 5.67 ^c^
MON (%)	13.8 ± 3.21	12.42 ± 2.21	13.8 ± 1.81	16.9 ± 3.34	12.4 ± 2.87
GRA (10^3^/µL)	3.98 ± 0.36 ^a^	2.34 ± 0.32 ^b^	3.32 ± 0.28 ^c^	3.46 ± 0.31 ^d^	3.56 ± 0.23 ^e^
RBC (10^3^/µL)	2.23 ± 0.13 ^a^	2.78 ± 0.23 ^b^	2.56 ± 0.34 ^c^	2.21 ± 0.19 ^a^	1.89 ± 0.21 ^d^
HGB (g/dL)	6.28 ± 1.08 ^a^	9.53 ± 0.68 ^b^	9.83 ± 0.72 ^b^	8.54 ± 0.34 ^c^	8.34 ± 0.56 ^c^
HCT (%)	26.5 ± 1.21	27.8 ± 1.89	25.6 ± 2.21	26.2 ± 2.31	27.2 ± 1.16
MCV (µm^3^)	125.3 ± 2.29	120.3 ± 3.18	125.7 ± 2.31	121.4 ± 3.32	126.8 ± 4.24
MCH (pg)	38.3 ± 3.18 ^a^	44.5 ± 2.43^b^	40.1 ± 2.56 ^c^	38.7 ± 2.45 ^a^	40.3 ± 3.78 ^c^
MCHC (g/dL)	28.7 ± 4.52 ^a^	36.4 ± 3.86 ^b^	35.2 ± 3.12 ^b^	34.3 ± 2.86 ^c^	33.2 ± 3.42 ^c^
RDW (%)	7.6 ± 0.45 ^a^	5.3 ± 0.34 ^b^	5.4 ± 0.28 ^b^	7.2 ± 1.08 ^a^	7.5 ± 0.86 ^a^
PLT (10^3^/µL)	42.3 ± 2.42 ^a^	30.2 ± 3.46 ^b^	32.5 ± 2.86 ^b^	31.7 ± 3.48 ^b^	39.4 ± 2.68 ^a^
MPV (µm^3^)	7.12 ± 0.78 ^a^	6.32 ± 1.34 ^b^	6.48 ± 0.86 ^b^	5.54 ± 0.98 ^c^	5.48 ± 0.72 ^c^
PCT (%)	0.02 ± 0.01	0.02 ± 0.01	0.01 ± 0.01	0.02 ± 0.01	0.01 ± 0.01
PDW (%)	7.68 ± 0.82 ^a^	9.32 ± 1.44 ^b^	7.42 ± 0.68 ^c^	9.54 ± 0.76 ^b^	9.42 ± 0.68 ^b^

Note: Different superscripts showed significant differences at *p* < 0.05. Abbreviation: WBC = white blood cell, LYM = lymphocytosis, MON = monocytes, GRA = granulocytosis, RBC = red blood cell, HGB = haemoglobin, HCT = hematocrit, MCV = mean corpuscular volume, MCH = mean corpuscular haemoglobin, MCHC = mean corpuscular haemoglobin concentration, RDW = red cell distribution width, PLT = platelet, MPV = mean platelet volume, PCT = procalcitonin, PDW = platelet distribution width.

**Table 6:** Blood chemical profiles of experimental fish (*n* = 3). Data expressed as mean ± standard deviation.

Blood Chemical Profiles	T1	T2	T3	T4	T5
ALB (g/dL)	0.74 ± 0.12 ^a^	1.12 ± 0.10 ^b^	1.14 ± 0.15 ^b^	1.13 ± 0.17 ^b^	1.21 ± 0.13 ^b^
GLOB (g/dL)	1.98 ± 0.14 ^a^	2.34 ± 0.10 ^b^	2.45 ± 0.08 ^b^	2.41 ± 0.16 ^b^	2.56 ± 0.06 ^b^
TP (g/dL)	2.98 ± 0.20 ^a^	3.34 ± 0.24 ^b^	3.45 ± 0.38 ^b^	3.41 ± 0.42 ^b^	3.56 ± 0.54 ^b^
BUN/urea (mg/dL)	3.46 ± 0.18	3.56 ± 0.12	3.64 ± 0.46	3.34 ± 0.34	3.68 ± 0.24
Crea (mg/dL)	0.14 ± 0.03	0.13 ± 0.05	0.12 ± 0.05	0.13 ± 0.01	0.12 ± 0.02
ALKP (µ/L)	11.24 ± 0.52	12.34 ± 0.51	11.18 ± 0.64	12.26 ± 0.74	12.17 ± 0.48
ALT (µ/L)	13.36 ± 1.48 ^a^	14.28 ± 2.32 ^a^	20.76 ± 4.68 ^b^	21.78 ± 3.78 ^b^	21.22 ± 5.64 ^b^
AST (µ/L)	68.18 ± 4.82 ^a^	69.46 ± 5.36 ^a^	84.62 ± 6.42 ^b^	88.72 ± 6.24 ^b^	86.24 ± 7.48 ^b^
GGT (µ/L)	0.98 ± 0.13	0.96 ± 0.23	0.95 ± 0.12	0.94 ± 0.14	0.97 ± 0.24
GLU (mg/dL)	56.32 ± 3.42 ^a^	70.42 ± 4.82 ^b^	71.68 ± 5.62 ^b^	72.14 ± 3.21 ^b^	81.38 ± 3.12 ^c^
CHOL (g/dL)	12.26 ± 0.14 ^a^	7.22 ± 0.32 ^b^	8.22 ± 0.32 ^c^	9.43 ± 0.42 ^d^	9.86 ± 0.23 ^d^
TBIL (mg/dL)	0.13 ± 0.02	0.13 ± 0.01	0.12 ± 0.02	0.12 ± 0.02	0.13 ± 0.01

Note: Different superscripts showed significant differences at *p* < 0.05. Abbreviation: ALB = Albumin, GLOB = globulin, TP = total protein, BUN = blood urea nitrogen, Crea = creatinine, ALKP = alkaline phosphatase, ALT = alanine aminotransferase, AST = aspartate aminotransferase, GGT = gamma glutamyltransferase, GLU = glucose, CHOL = cholesterol, TBIL = total bilirubin.

## Data Availability

Not applicable.
